# Phytochemical baicalin potentially inhibits Bcl-2 and VEGF: an *in silico* approach

**DOI:** 10.3389/fbinf.2025.1545353

**Published:** 2025-02-19

**Authors:** Vikas Sharma, Arti Gupta, Mohini Singh, Anshul Singh, Anis Ahmad Chaudhary, Zakir Hassain Ahmed, Salah-ud-din Khan, Sarvesh Rustagi, Sanjay Kumar, Sandeep Kumar

**Affiliations:** ^1^ Sharda School of Pharmacy, Sharda University, Greater Noida, Uttar Pradesh, India; ^2^ Metro College of Health Science and Research, Greater Noida, Uttar Pradesh, India; ^3^ Lloyd Institute of Management and Technology, Greater Noida, Uttar Pradesh, India; ^4^ Department of Life Sciences, Sharda School of Basic Sciences and Research, Sharda University, Greater Noida, India; ^5^ Sharda School of Allied Health Sciences, Sharda University, Greater Noida, Uttar Pradesh, India; ^6^ Department of Biology, College of Science, Imam Mohammad Ibn Saud Islamic University (IMSIU), Riyadh, Saudi Arabia; ^7^ Department of Mathematics and Statistics, College of Science, Imam Mohammad Ibn Saud Islamic University (IMSUI), Riyadh, Saudi Arabia; ^8^ Department of Biochemistry, College of Medicine, Imam Mohammad Ibn Saud Islamic University (IMSIU), Riyadh, Saudi Arabia; ^9^ Department of Food Technology, School of Applied and Life Science, Uttaranchal University, Dehradun, Uttarakhand, India; ^10^ DST-FIST Lab, Sharda University, Greater Noida, Uttar Pradesh, India

**Keywords:** cancer, phyto-analogs, baicalin, VEGF, anticancer

## Abstract

**Background:**

The rising prevalence of cancer cells exhibits uncontrolled growth and invasive and aggressive properties, leading to metastasis, which poses a significant challenge for global health. Central to cancer development are proteins such as NF-kB, p53, VEGF, and BAX/Bcl-2, which play important roles in angiogenesis, cell apoptosis regulation, and tumor growth.

**Methodology:**

This *in silico* study evaluates the activity of six different natural as well as novel therapeutic strategies against cancer. Using a computational approach, i.e., virtual screening, molecular docking, and molecular dynamics (MD) simulations, the binding affinities and interactions of selected phytochemicals with cancer-specific proteins were analyzed. Key criteria for selection included binding affinity, molecular stability, and pharmacokinetic and toxicological properties. Post-selection, dynamics of ligand–protein interactions were further examined through MD simulations conducted using Desmond-Maestro 2020-4 on a Linux-based HP Z2 workstation, providing an insight into the conformational changes in the stability of the inhibitor–protein complexes. This was complemented by ADMET predictions to assess pharmacokinetics and toxicological profiles.

**Results:**

Our findings reveal that out of six phytochemicals, baicalin exhibited the most promising results, with docking scores of −9.2 kcal/mol and −9.0 kcal/mol against Bcl-2 and VEGF receptors, respectively. The MD simulation (100 ns) confirmed the stability of baicalin–protein interactions, supported by hydrophobic interactions and intermolecular hydrogen bonds. The RMSD and RMSF values of baicalin exhibit an acceptable global minimum (3.5–6 Å) for p53, VEGF, and BAX/Bcl-2.

**Conclusion:**

This study highlights the potential of baicalin, a phytochemical known for anti-cancerous, anti-apoptotic, and anti-proliferative properties, as a promising candidate for cancer treatment. Further exploration and validation of its inhibitory mechanisms could open a promising avenue for therapeutic approaches in oncology.

## Graphical Abstract

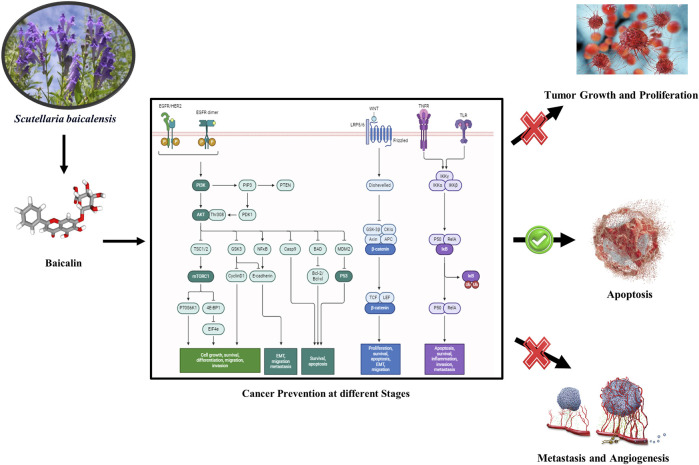

## 1 Introduction

Cancer is characterized by uncontrolled and abnormal proliferation of cells, which can invade and metastasize to different body organs (Shuvalov et al., 2023). There are different hallmarks for the development of the cancer, which includes increased cellular proliferation and growth regulators, decreased apoptosis, induced angiogenesis, replicative immortality, and metastasis (Ardies, 2003; Hanahan and Weinberg, 2011; Krishnamurthy and Kurzrock, 2018).

Cancer is a leading cause of death worldwide, and the number of patients continues to increase (Clark, 1991). As per the classification of Global Cancer Observatory (GCO) and World Health Organization (WHO), cancer which affects healthy human beings can be classified into 100 different types (Siegel et al., 2024). Bronchiole and lung cancer exhibits a higher rate of mortality, followed by breast and stomach cancer (Sharma et al., 2024). Over 1.9 million new cases of cancer have been diagnosed so far in the United States, where prostate cancer is prevalent in the male population (29%) and breast cancer is prevalent in the female population (31%) of the United States (Hu et al., 2013; Siegel et al., 2020). According to international organizations such as the WHO and GCO, global cancer burden is staggering, with over 19 million individuals currently battling the disease. Alarmingly, this number is projected to surge to over 28 million by 2040, highlighting the urgent need for innovative treatments and preventive measures to combat this growing health crisis (Sung et al., 2021). It is expected that over 9.5 million new cases per year in Asia might increase by 59% and the highest increase of approximately 89.1% is expected in the African continent by 2040 (Dizon and Kamal, 2024; Mathur et al., 2020; Spagnuolo et al., 2015).

Despite significant efforts, cancer exhibits high mortality worldwide (Ai et al., 2017). Different conventional methods like allopathic medications, surgery, chemotherapy, and radiotherapy are used for treatment and management of cancer (Safarzadeh et al., 2014; Ullrich et al., 2019). Despite their benefits, current cancer therapies are hindered by significant limitations and drawbacks. A substantial number of patients are diagnosed at an advanced stage, making surgical intervention no longer viable due to delayed diagnosis and other contributing factors. Furthermore, conventional treatments like chemotherapy and radiotherapy often lead to debilitating side effects, including fatigue, pain, gastrointestinal distress, nausea, vomiting, and alopecia, significantly impacting patients’ quality of life (Pereira et al., 2012). Moreover, cancer cells can gradually develop resistance to chemotherapy and radiotherapy (Desai et al., 2008).

Cancer prevention is highly important, and herbal remedies can play a significant role in this regard. Herbal drugs such as fisetin, berberis, bloodroot, EGCG, and sanguinarine have shown potential in fighting cancer (Kumar, 2007; Sharma et al., 2018). Ayurvedic superfoods like amla and garlic are known for their anti-cancer properties. These herbs not only have the potential to minimize the cancer reoccurrence and chances of development of various cancer types but also enhance overall health (Bose et al., 2020). Although herbal supplements have served as adjunctive therapies, they should not supplant conventional cancer treatments. Regular screenings and self-examinations remain essential for early detection and effective management (Fujiki et al., 2018; Schabath and Cote, 2019). Thus, integrating herbal remedies into a healthy lifestyle can contribute to cancer prevention and improve the quality of life for individuals and communities (Lim and Wang, 2020; Singh et al., 2022). In this study, we have selected six different phytochemicals based on literature reviews. Phytochemicals, including baicalin, EGCG, β-carotene, fisetin, sanguinarine, and 6-gingerol, have demonstrated *in vitro* anti-cancer activities. Specifically, two phytochemicals (e.g., 6- gingerol and fisetin) have been reported to target cancer cell proliferation (Farombi et al., 2020; Suh et al., 2009), two (e.g., sanguinarine and β-carotene) have been shown to induce apoptosis (Kavalappa et al., 2019; Kuttikrishnan et al., 2019), and two (e.g., baicalin and EGCG) have been found to inhibit angiogenesis (Shehatta et al., 2022; Wang et al., 2018). These phytochemicals were selected based on their reported potency, minimal effective doses, and distinct mechanisms of action within each category. Consequently, they were chosen for *in silico* analysis to further investigate their potential in cancer prevention and treatment.

## 2 Materials and methods

### 2.1 Preparation of the target protein and library construction

Signaling molecules involved in cancer progression (NF-kB, p53, VEGF, and Bcl-2) are selected for *in silico* studies that are proactively involved in cancer development mechanisms (Baek et al., 2016; Chiarugi et al., 1999; Chouhan et al., 2024). The three-dimensional (3D) protein structures were downloaded using the RCSB PDB directory (Research Collaboratory for Structural Bioinformatics Protein Data Bank (Burley et al., 2023). PDB IDs of targeted proteins are 7EAL, 3LGF, 3QTK, and 2W3L (Hong et al., 2021; Roy et al., 2010; Mandal and Kent, 2011; Porter et al., 2009) for NF-kB, p53, VEGF, and Bcl-2, respectively. Protein structures were prepared in UCSF Chimera for the screening of compounds (Pettersen et al., 2004).

### 2.2 Ligand preparation

A comprehensive literature review on herbal anti-cancer phytomolecules was conducted using databases, including Google Scholar, ScienceDirect, and PubChem (Kim et al., 2024; Vélez-Vargas et al., 2023). The 3D structures of the compounds were retrieved from the PubChem database in Standard Data File (SDF) format. These structures were then converted to Protein Data Bank (PDB) format using Progenesis SDF Studio software (Nguyen Thi Thu et al., 2023). The prepared ligands were then docked against selected targets using AutoDock Vina 1.5.6 program (Chen et al., 2024).

### 2.3 Active site detection

We have identified active sites using the Computed Atlas of Surface Topography of proteins (CASTp) webserver for identifying active and binding pockets of any receptor accessible at (Nguyen Thi Thu et al., 2023; Tian et al., 2018; Tian et al., 2018). These active sites identified by CASTp have been added into the active site of the macromolecule section of PyRx (Dallakyan and Olson, 2015; Hossain et al., 2023).

### 2.4 Ligand–target interaction

We have used PyRx software for molecular docking studies, which follows algorithms of AutoDock 4, AutoDock Vina, and Python (Dallakyan and Olson, 2015; Manoharan et al., 2023). The identified active sites were used for grid generation in the target protein (Parihar et al., 2023). PyRx employed AutoDock Vina to generate multiple ligand-binding poses, ranked based on their binding affinities. A threshold binding energy of −6 kcal/mol is considered indicative of active drugs (Manoharan et al., 2023).

The resultant files were analyzed using BIOVIA Discovery Studio to elucidate the binding interactions, orientations, and energy levels of various ligands with target proteins (Ghelichkhani et al., 2023; Parihar et al., 2024). Notably, hydrogen bonding significantly influences docking scores, thereby impacting structural characteristics of novel drug discovery and their development (Chen et al., 2022). The selected compounds were further analyzed by molecular dynamics simulation (MDS) to assess the flexibility and stability of complexes of different ligands and proteins (S. Kumar et al., 2023).

### 2.5 Molecular dynamics simulation

Molecular dynamics simulations (MDS) were performed on the complexes (phytochemical and proteins, NF-kB, p53, VEGF, and Bcl-2) with the best docking score Bcl-2 to examine their intermolecular interactions and stability. The simulations were conducted using the Desmond-maestro 2020-4 academic package on a Linux-based HP Z2 workstation (Bowers, K. J. et al., 2006; Dai et al., 2016). The complexes formed between the lead molecules and NF-kB, p53, VEGF, and Bcl-2 targets were set up in all directions (20 Å × 20 Å x 20 Å) to allow sufficient space for fluctuations (Shah et al., 2023). The system was then neutralized using optimal sodium^+1^/chloride^−1^ counter particles, and the TIP4P (transferable intermolecular potential with 4 points) model was used to achieve optimum solvation properties for the MDS process (Dwivedi et al., 2020). MDS was performed using a combination of advanced algorithms, including the Nose–Hoover thermostat and Martyna–Tobias–Klein method, to mimic the behavior of the entire system across a temperature range of 100 K–300 K and a constant pressure of 1.0 atm (Abdallah et al., 2018; Fu et al., 2012; Li et al., 2021; Toma et al., 1995) The velocities were measured using the Berendsen algorithm, and a 9 Å cutoff radius was applied to the Lennard–Jones potential to efficiently model van der Waals interactions (Desai et al., 2008; Parihar et al., 2024; Rabaan et al., 2024; Zhao et al., 2023). This simulation framework enabled a comprehensive investigation of the system’s thermodynamic properties, structural dynamics, and molecular interactions under various temperature conditions, providing valuable insights into its behavior and properties (Fei, 2023; Kusakabe et al., 2023; Shah et al., 2023; Xiang et al., 2022;Yang et al., 2022).

All data calculations were performed using the OPLS2005 force field for all atoms (Verma et al., 2021). The conformations or trajectories were obtained from MDS outcomes for each potential docked ligand complexed with NF-kB, p53, VEGF, and Bcl-2 proteins and were analyzed to measure the root mean square deviation and root mean square fluctuation and to outline different relatable interactions between the ligand and the targeted protein/receptor (Singh et al., 2021).

### 2.6 ADMET analysis of baicalin

The ADMET analysis of Baicalin was performed using ADMETlab 3.0, which evaluated its absorption, distribution, metabolism, excretion, and toxicity properties (Nguyen Thi Thu et al., 2023). The analysis included predictions of Caco-2 permeability, human intestinal absorption, bioavailability, plasma protein binding, blood–brain barrier penetration, volume of distribution, CYP enzyme interactions, plasma clearance, and half-life (Nguyen Thi Thu et al., 2023). Additionally, the analysis assessed the compound’s toxicity risks, including hERG blockers, carcinogenicity, Ames mutagenicity, skin sensitization, and drug-induced liver injury (Soukaina et al., 2023).

## 3 Results

### 3.1 Structure-based virtual screening

**FIGURE 1:**
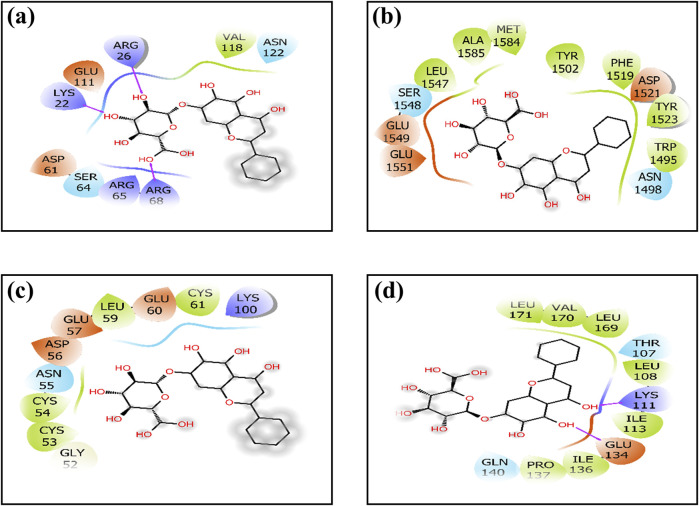
Two-dimensional interactions of baicalin with cancer progression targets **(A)** Bcl-2, **(B)** p53, **(C)** VEGF, and **(D)** NF-kB. The creation of H-bonds in docked poses is indicated by pink arrows; correspondingly negative residues in red, green, and blue; hydrophobic interactions in green; polar residues in blue; gray residues indicate glycine; and salt bridge interactions have been notified in red and blue.

Molecular docking was performed for different ligands against the identified target proteins including NF-kB, p53, VEGF, and Bcl-2. The docking score for the interactions ranged from −9.2 kcal/mol to −4.9 kcal/mol. Baicalin (64982) showed the best docking scores against all the four targets compared to other phytochemicals (specifically, in targets of proliferation/metastasis). It exhibited a docking score of −9.2 Kcal and −9.0 Kcal with Bcl-2 and VEGF receptor, respectively (Table 2; Figure 1). The binding poses of baicalin with its targets, NF-kB (64982-7EAL), p53 (64982-3LGF), VEGF (64982-3QTK), and Bcl-2 (64982-2W3L), were evaluated based on the maximum free binding energy values, and the most favorable poses were chosen for subsequent investigation and analysis (Table 1).

**TABLE 1:** PubChem IDs of selected phyto-analogs.

PubChem IDs	Active phyto-analog
64982	Baicalin
65064	EGCG
5280489	β-Carotene
5281614	Fisetin
5154	Sanguinarine
442793	6-Gingerol

**TABLE 2:** Docking scores of selected natural phytomolecules with NF-kb, p53, BCl-2, and VEGF targets of cancer development and progression.

NPs/receptor	PubChem ID	Docking score (Kcal/mol)			
		NF-κB	p53	Bcl2	VEGF
Fisetin	5281614	−6.4	−6.3	−7.7	−7.8
β-Carotene	5280489	−6.3	−6.9	−8.2	−8.4
EGCG	65064	−5.5	−7.2	−8.0	−7.7
6-Gingerol	442793	−4.9	−6.6	−5.7	−6.0
Sanguinarine	5154	−6.7	−7.7	−8.8	−9.0
Baicalin	64982	−6.1	−7.6	−9.2	−9.0

### 3.2 Docked pose interactions

**FIGURE 2:**
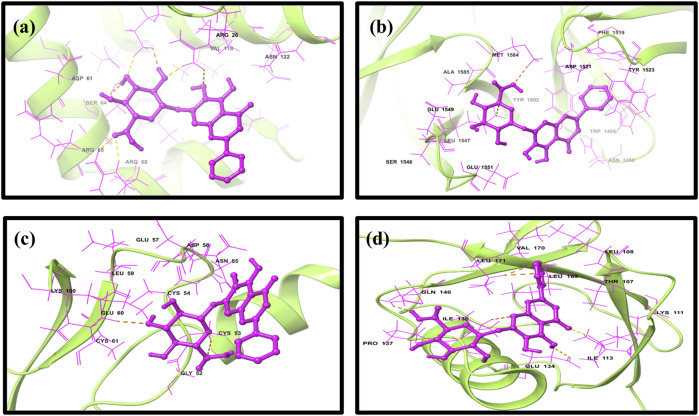
Three-dimensional docked complex pose of selected phytomolecule baicalin with cancer progression targets **(A)** Bcl-2, **(B)** p53, **(C)** VEGF, and **(D)** NF-kB.

To elucidate complex stability, an in-depth analysis of molecular interactions was performed for each docked complex. The docked complex of 64982-2W3L (baicalin-Bcl-2) exhibited formation of three hydrogen bonds with LYS^22^, ARG^26^, and ARG^68,^ as depicted in Figures 1A, 2A. It is responsible for increasing the strength and stability of ligand–receptor binding. Studies indicate that higher the number of hydrogen bonds, higher will be the interaction strength between the ligand and target. The complex also showed one Bcl-2 hydrophobic interaction at VAL^118^. Another complex 64982-3LGF (baicalin-p53) showed seven hydrophobic interactions at LEU^**1547**^, ALA^**1585**^, MET^**1584**^, TYR^**1502**^, PHE^**1519**^, TYR^**1523**^, and TRP^**149**5^ (Figures 1B, 2B), and the docked complex of 64982-3QTK (baicalin-VEGF) showed four hydrophobic interactions at CYS^**53**^, CYS^**54**^, LEU^**59**^, and CYS^**61**^ (Figures 1C, 2C). Furthermore, 64982-7EAL (baicalin-NF-kB) exhibited two hydrogen bonds at LYS^111^ and GLU^134^ (Figures 1D, 2D) and seven hydrophobic interactions at LEU^108^, ILE^113^, ILE^136^, PRO^137^, LEU^171^, VAL^170^, and LEU^169^ (Figures 1D, 2D). No hydrogen bonds were observed for the docked complex 64982-3LGF (baicalin-p53) and 64982-3QTK (baicalin-VEGF) (Figures 1C, 2C). In addition to the primary interactions, residues sharing similar characteristics and engaging in secondary interactions, including hydrophobic, polar, and negatively charged interactions, are highlighted in Figure 1 and detailed in Table 3. These supplementary interactions contribute significantly to the overall stability and specificity of the complex.

**TABLE 3:** Molecular interaction profiling of the selected phytomolecule baicalin with cancer progression targets A) BCl-2, B) p53, C) VEGF, and D) NF-kB.

Sr No.	Baicalin complex with	Hydrogen bond	Hydrophobic interaction	Polar	Negative
1	7EAL	LYS^111^, GLU^134^	LEU^108^, ILE^113^, ILE^136^, PRO^137^, LEU^171^, VAL^170^, and LEU^169^	GLU^134^	GLU^134^
2	3LGF	-	LEU^1547^, ALA^1585^, MET^1584^, TYR^1502^, PHE^1519^, TYR^1523^, and TRP^1495^	GLU^1549^, GLU^1551^, and ASP^1521^	-
3	3QTK	-	CYS^53^, CYS^54^, LEU^59^, and CYS^61^	ASP^56^, GLU^57^, and GLU^60^	LYS^100^
4	2W3L	LYS^22^, ARG^26^, and ARG^68^	VAL^118^	ASP^61^ and GLU^111^	LYS^22^, ARG^26^, ARG^65^, and ARG^68^

**TABLE 4:** Toxicological and safety profile of baicalin.

A/D/M/E/T	Property	Value	Comment
Absorption	Caco-2 permeability	−6.76	Optimal: higher than −5.15 Log unit
	MDCK	0.0	■ low permeability: <2 × 10^−6^ cm/s
	Permeability		■ medium permeability: 2–20 × 10^−6^ cm/s
	PAMPA	+++	
Distribution	PPB	90.1%	■ Drugs with high protein-bound may have a low therapeutic index
	VDss	0.603	■ Volume distribution
	BBB	0.012	Blood–brain barrier penetration
	Fu	6.9%	■ The fraction unbound in plasms
Metabolism	CYP1A2 inhibitor	**1**	■ Category 1: inhibitor; Category 0: non-inhibitor
	CYP1A2 substrate	**1**	■ Category 1: substrate; Category 0: non-substrate
	CYP2C19 inhibitor	**1**	■ Category 1: inhibitor; Category 0: non-inhibitor
	CYP2C19 substrate	**1**	■ Category 1: substrate; Category 0: non-substrate
	CYP3A4 inhibitor	**1**	■ Category 1: inhibitor; Category 0: non-inhibitor
	CYP2C8 inhibitor	**1**	■ Category 1: inhibitor; Category 0: non-inhibitor
	HLM	0.212	■ human liver microsomal (HLM) stability
Excretion	CL plasma	1.736	■ The unit of predicted CL plasma penetration is mL/min/kg. >15 mL/min/kg: high clearance; 5–15 mL/min/kg: moderate clearance; <5 mL/min/kg: low clearance
	T1/2	3.397	■ The unit of predicted T1/2 is hours
Toxicity	Hepatotoxicity	NO	
	Genotoxicity	NO	
	Skin sensitization	NO	
	Eye irritation	NO	
	Ototoxicity	NO	

### 3.3 MD simulation analysis

The stability of molecularly docked poses and the outline of their intermolecular interactions can be examined through MDS. In this study, we conducted a 100-ns explicit solvent MDS to evaluate the stability and intermolecular interaction between the baicalin docked with NF-kB, p53, VEGF, and Bcl-2. MDS enables a comprehensive understanding of the molecular recognition and binding affinity between the ligand and the target protein.

### 3.4 Root mean square deviation analysis

**FIGURE 3:**
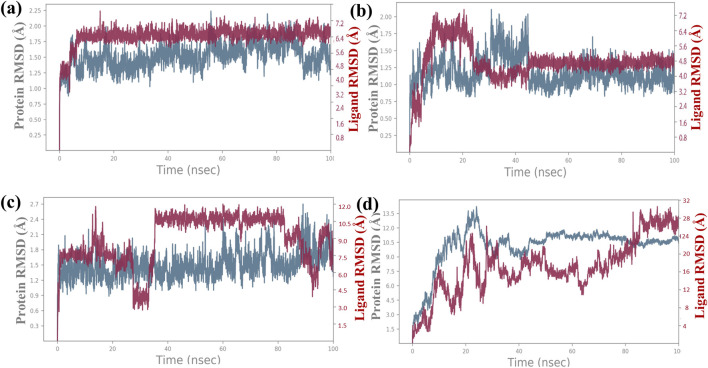
RMSD plot for backbone elements of the targeted proteins that was chosen, baicalin as the ligand molecule, and the atoms of **(A)** Bcl-2, **(B)** p53, **(C)** VEGF, and **(D)** NF-kB. The trajectories of multiple docked complexes have been developed from 100.0 nanoseconds of the molecular dynamics simulation study duration.

The root mean square deviation (RMSD) was calculated to assess the conformational alterations in both the protein and ligand upon complex formation. In Figure 3, we observe the RMSD values for proteins and ligands in the docked molecules. Remarkably, the RMSD value of the Cα atom present in the protein remains consistently below 3 Å across all complexes. This stability implies that the protein experiences minimal fluctuation upon ligand binding, maintaining a consistent conformation. Furthermore, it suggests robust binding between the ligand and the protein, preventing significant structural changes. Overall, the protein–ligand complex exhibits remarkable stability. The ligand RMSD was determined by aligning the different Cα atom present in structures of proteins. The stability of the atoms in the docked proteins Bcl-2, p53, VEGF, NF-kB, and baicalin was evaluated in a 100-ns simulation using RMSD and RMSF. The RMSD for the alpha-carbon atom in Bcl-2, p53, and VEGF proteins demonstrated satisfactory stability (less than 3 Å) all over the period of the simulation experiment, as shown in Figures 3A–C, and p53 and VEGF showed a state of equilibrium (<2 Å) from 0 to 100 ns. RMSD for the alpha-carbon atom in the NF-kB protein showed fluctuations >10 Å, as depicted in Figure 3D. These data were further supported by the RMSF values.

Additionally, a detailed analysis was performed on the RMSD values for the ligand-fit protein in each docked complex, examining them individually to assess the accuracy and reliability of the docking results. The RMSD analysis of the baicalin–Bcl-2 complex showed an acceptable global minimum (3.5–6 Å) and was found to maintain equilibrium (Figure 3A). The RMSD analysis of the baicalin–p53 complex showed a comparatively minor deviation (6 Å) and eventually reached a significant equilibrium (started with 3 Å up to 10 ns, then increased to 7 Å up to 2 0 ns, and thereafter from 20 ns to 100 ns showed stability at approximately 5 Å) as shown in Figure 3B. The RMSD analysis of the VEGF–baicalin complex displayed a stable equilibrium (4 Å) with minor deviations at 16 and 22 ns (Figure 3C). The complex of baicalin with NF-kB has not shown significant ligand fit protein interactions, as depicted in Figure 3D, and thus concluded to be a less stable complex.

### 3.5 Root mean square fluctuation analysis

The RMSF plot exhibits deviation of the protein backbone atoms in MDS, which provides important information flexibility and the stability of the protein structures, along with the binding behavior of small molecules or ligands. The RMSD plot of baicalin with p53, VEGF, and Bcl-2 exhibits stability of the docked complexes. This finding was further corroborated by the RMSF analysis of each of the selected complexes, which highlighted the significance of RMSF values in quantifying local oscillations between protein chains and ligand molecules, providing valuable insights into their dynamic interactions. A low RMSF value indicates a more stable protein structure, while a high RMSF value indicates a more flexible structure**.** To determine local structure fluctuations, amino acid residues of the p53, VEGF, Bcl-2, and NF-kB proteins, as well as the atoms of the docked compound, were examined. The RMSF value of less than 4 Å was observed across all simulations, except for NF-kB (Figure 5). Remarkably, the protein residues in all the complexes exhibited acceptable RMSF values, with the exception of the C- and N-terminal regions. From the protein RMSF analysis, it can be inferred that the receptor proteins (p53, VEGF, and Bcl-2) remain in a stable state without any conformational changes during the binding of the natural compound, baicalin within the MD simulation time frame.

**FIGURE 4:**
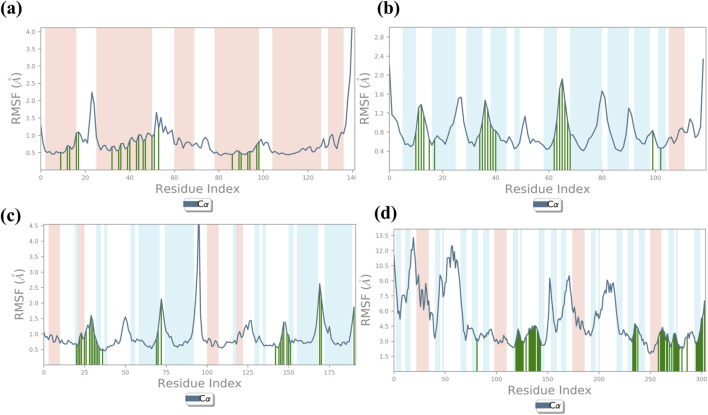
RMSF plot generated for baicalin with cancer targets **(A)** Bcl-2, **(B)** p53, **(C)** VEGF, and **(D)** NF-kB, during the 100-ns molecular dynamics simulation interval. Regions shaded in red denote areas of elevated RMSF values, signifying enhanced flexibility or increased fluctuation dynamics within those specific regions.

**FIGURE 5:**
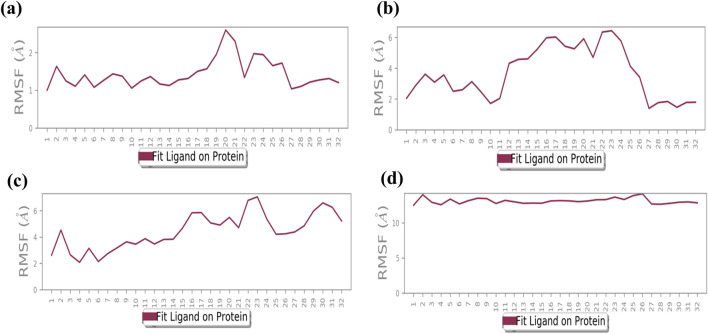
Illustrates the average root mean square fluctuation (RMSF) values of every atom/residue with protein and ligand molecules, calculated over a 100-ns molecular dynamics (MD) simulation for the following cancer targets: **(A)** Bcl-2, **(B)** p53, **(C)** VEGF, and **(D)** NF-kB.

Interestingly, all amino acid residues of p53, VEGF, and Bcl-2 protein complexes (Figures 4A–C, 5A–C) provided permissible RMSF values, with the exception of NF-kB (Figures 4D, 5D). The RMSF analysis of the ligand molecule revealed stable binding with minimal residual fluctuations (less than 4.5 Å) across all complexes, except for NF-kB (Figures 4A–D). When combined with the RMSD data of all the docked complexes, these findings support the potential integration of baicalin with anti-cancer targets p53, VEGF, and Bcl-2.

### 3.6 Protein–ligand interaction mapping

**FIGURE 6:**
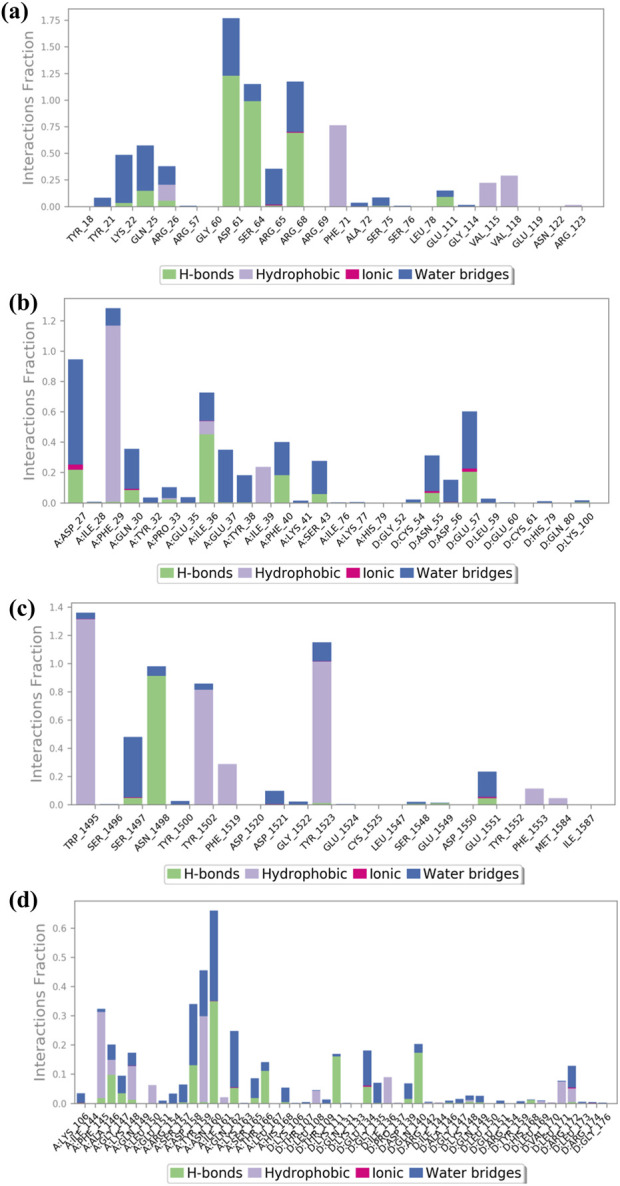
Interaction of ligand–protein mapping of selected phytomolecule baicalin with cancer progression targets **(A)** Bcl-2, **(B)** p53, **(C)** VEGF, and **(D)** NF-kB, as the data outcomes from the MD simulation study for 100 nano seconds.

**FIGURE 7:**
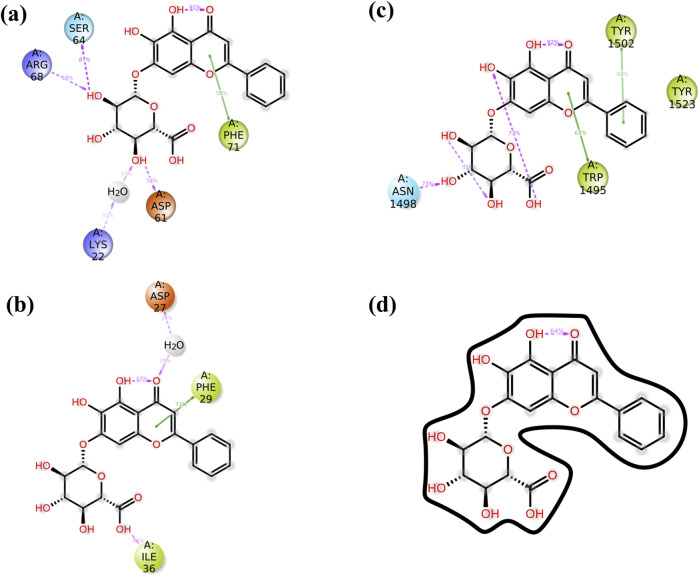
The schematic diagram provides a detailed view of the atomic interactions between baicalin and various cancer target receptors, namely **(A)** 2W3L (Bcl-2), **(B)** 3LGF (p53), and **(C)** 3QTK (VEGF), and **(D)** 7EAL (NF-kB). The diagram focuses on a trajectory for 100 nanoseconds and has shown molecular interactions which were present for over 30% of the overall period of the simulation.

The complexes of NF-kB, p53, VEGF, and Bcl-2 proteins with baicalin can be further analyzed to elucidate the protein–ligand binding interaction landscape, encompassing the dynamics of hydrogen bonding, hydrophobic interactions, water-mediated bridges, and ionic contacts over the course of a 100-ns MD simulation. Docked complex baicalin-Bcl-2 shows hydrogen bond formation at GLY^60^, ASP^61^, ARG^65^, LEU^78^, LYS ^22^, GLN^25^, TYR ^21^, and GLN^25^ along with water bridges (Figure 6A). In protein–ligand interaction mapping, the baicalin–2W3L docked complex has shown strong hydrogen bonding at TYR^21^, LYS^22^, GLN^25^, GLY^60^, ASP^61^, ARG^65^, and GLU^111^. Additionally, independent water bridges have been mapped at TYR^18^, PHE^71^, ALA^72^, and GLU^111^. It also shows the presence of hydrophobic interactions at GLY^114^, VAL^115^, and ARG^69^. A hydrophobic interaction also has been found at GLN^25^ along with hydrogen bonds and water bridges. An ionic interaction was found at SER^64^ along with water bridges (Figures 6A, 7A). Baicalin–p53 docked complex exhibited strong hydrophobic interactions at TRP^1495^, TYR^1502^, PHE^1519^, TYR^1523^, PHE^1533^, and MET^1584^. Water bridges along with hydrogen bonds were formed at TRP^1495^, TYR^1502^, TYR^1500^, PHE^1519^, and TYR^1523^, whereas independent water bridges were observed at ASP^1521^, GLY^1522^, and TYR^1500^. Hydrogen bonds were mapped in this complex at ASN^1498^, SER^1497^, and GLU^1551^ (Figures 6B, 7B).

The protein–ligand docked complex of baicalin–VEGF showed hydrogen bonding at ASP^27^, GLN^30^, PRO^33^, ILE^36^, PHE^40^, and SER^43^ in chain A, while ASN^55^ and GLU^57^ in chain D of protein (along with water bridges). Fewer distinct water bridges have also been observed at TYR^32^, GLU^35^, GLU^37^, TYR^38^, and LYS^41^ in chain A of the protein, while CYS^54^, ASP^56^, LEU^59^, HIS^79^, and LYS^100^ in chain D of 3QTK protein. This interaction has also shown hydrophobic interactions at PHE^79^, ILE^36^, and ILE^39^ in chain A of the VEGF protein. Ionic interactions were mapped at ASP^27^, GLN^30^, ASN^55^, and GLU^57^ (Figures 6C, 7C). The baicalin–NF-kB docked complex has also been mapped for drug–protein interactions, which showed hydrogen bond formation at PHE^145^, ALA^146^, GLY^147^, ASP^158^, ASN^160^, GLN^162^, SER^165^, and THR^166^ in chain A along with water bridges and hydrophobic interactions, while chain D of protein showed strong hydrogen bond formations at LYS^111^, ASP^139^, and ARG^140^. Hydrophobic interactions in the baicalin–NF-kB docked complex have been mapped at PHE ^145^, LYS^148^, TYR^159^, ILE^161^, LEU^108^, ILE^136^, LEU^171^, and ARG^172^ along with water bridges (Figures 6D, 7D). Ionic interaction was observed at GLU^134^.

### 3.7 ADMET outcomes

The ADMET analysis of baicalin revealed concerns regarding its absorption, distribution, and toxicity properties. Specifically, baicalin showed low Caco-2 permeability (−6.76) and moderate MDCK permeability (0.0), indicating potential absorption issues. Distribution analysis revealed high protein binding (90.1% PPB) and a relatively low volume of distribution (0.603 VDss). Baicalin was also predicted to be a substrate and inhibitor of various CYP enzymes, including CYP1A2, CYP2C19, and CYP3A4. Additionally, baicalin’s human liver microsomal stability was moderate (0.212), and its clearance was predicted to be moderate (1.736 mL/min/kg). However, baicalin was not predicted to exhibit hepatotoxicity, genotoxicity, skin sensitization, eye irritation, or ototoxicity (Table 4).

## 4 Discussion

The findings of this *in silico* study unequivocally demonstrate that baicalin possesses significant potential as a therapeutic agent for cancer treatment and prevention. Notably, baicalin exhibited low binding energy with key target proteins, including p53, VEGF, and Bcl-2, indicating strong interactions and potential inhibitory effects. Particularly, baicalin’s strong binding affinity (−9.2 kcal) with Bcl-2 suggests that it may induce apoptosis by targeting both intrinsic and extrinsic pathways (Huang et al., 2022; Li et al., 2021; Liu et al., 2003; S; Singh et al., 2021). This apoptotic-inducing effect is crucial for eliminating cancer cells and preventing tumor progression. Furthermore, baicalin’s interaction with VEGF may diminish its effects, thereby preventing metastasis and inhibiting angiogenesis, a critical step in cancer progression (Z. Hu et al., 2022; Shehatta et al., 2022). The RMSD and RMSF plot obtained through 100-ns MD simulation against docked complexes of baicalin and target proteins exhibited a strong interaction and stability, except for NF-kB. Strong hydrogen bond interactions of the baicalin–Bcl2 docked complex showed hydrogen bonds at GLY^60^, ASP^61^, and ARG^65^; strong hydrophobic interactions at TRP^1495^, TYR^1502^, PHE^1519^, TYR^1523^, and PHE^1533;^ and other bindings that stabilize baicalin on the target protein and thus have a potential to be used as an anti-cancerous agent (Kusakabe et al., 2023). Baicalin is also known for its anti-cancerous activity through *in vitro* studies performed in different cell lines, such as MCF-7, MDA-MB231, 3T3, and A-549. The interaction of baicalin with p53 indicates that it is effective in management of DNA damage or alterations (Parihar et al., 2024). Activation of p53 is associated with inhibition of the cyclin–CDK complex, which is involved in proliferation (Abdallah et al., 2018; Fu et al., 2012; Abdallah et al., 2018; Fu et al., 2012). Tumor suppressor gene, p53 has also been reported to play a crucial role in activation of BAX and inhibition of Bcl-2 in the apoptotic pathway via regulation of PUMA (p53 upregulated modulator of apoptosis) (Parihar et al., 2024; Zhao et al., 2023). Bax activation eventually results in the apoptosis of cancer (Toma et al., 1995). Baicalin also has shown direct effects on Bcl-2, another hallmark for initiating cancer cell death. Inhibition of Bcl-2 (p53-dependent and -independent) leads to the activation of a caspase-cascade system, resulting in an increased apoptotic rate. Interestingly, baicalin has inhibitory actions on VEGF as well (Kusakabe et al., 2023; Xiang et al., 2022; Zhao et al., 2023). VEGF is secreted in response to hypoxic conditions of a tumor to fulfil the requirement for nutrients and blood supply (Kumar Maurya and Lomte, 2022; Verma et al., 2021). VEGF is responsible for the formation of new blood vessels and metastasis via activation of VEGFR (Kumar Maurya and Lomte, 2022). These properties of baicalin promote it as a cancer therapeutic for cancer prevention-related research (S. Singh et al., 2021; Verma et al., 2021). Furthermore, use of baicalin with other drugs having inhibitory properties against NF-kB may result in development of a highly effective combination for anti-cancer therapy.

## 5 Conclusion

Cancer prevention involves resisting cancer development at its initial stages (NF-kB and p53), retarding angiogenesis (VEGF) and initiating cancer cell apoptosis (BAX/Bcl-2 and TNF-alpha). Eradicating this complex and debilitating disease has emerged as a significant challenge for researchers. Among all selected phytochemicals, baicalin demonstrated a promising docking score and was prioritized for MDS. This has shown efficacious binding with p53, VEGF, and Bcl-2, although its binding with NF-kB was comparatively weaker. Baicalin’s *in silico* activity highlights its potential as an anti-cancer agent. Its ability to interact with multiple targets involved in cancer progression suggests its multi-faceted mechanism of action. Furthermore, an ADMET analysis confirms baicalin’s favorable pharmacokinetic and safety profile, with minimal risk or hepatotoxicity, genotoxicity, or other systemic toxicities.

Although these computational studies, including molecular docking and MDS, provide valuable insights into baicalin’s molecular mechanisms, they also underscore the necessity for experimental validation. Rigorous *in vitro* and *in vivo* investigations are required to confirm the therapeutic potential of baicalin and elucidate its biological pathways. In conclusion, the findings of this research position baicalin as a promising candidate for cancer prevention. With further studies, it may contribute to the development of novel, more effective, and targeted strategies for cancer treatment, offering hope for improved patient outcomes.

## Data Availability

The original contributions presented in the study are included in the article/Supplementary Material; further inquiries can be directed to the corresponding authors.
